# Adipose-derived stem cell exosomes act as delivery vehicles of microRNAs in a dog model of chronic hepatitis

**DOI:** 10.7150/ntno.93064

**Published:** 2024-03-09

**Authors:** Ilaria Zanolla, Martina Trentini, Elena Tiengo, Federica Zanotti, Tommaso Pusceddu, Andrea Rubini, Giuseppe Rubini, Francesca Brugnoli, Danilo Licastro, Marco Debortoli, Lucia Gemma Delogu, Letizia Ferroni, Luca Lovatti, Barbara Zavan

**Affiliations:** 1 Department of Medical Sciences, University of Ferrara, 44121, Ferrara, Italy.; 2 Department of Translational Medicine, University of Ferrara, 44121, Ferrara, Italy.; 3 University of Bologna, 40126, Bologna, Italy.; 4 Ultravet Diagnostic, 40017, San Giovanni in Persiceto, Italy.; 5 AREA Science Park, 34149, Trieste, Italy.; 6 Department of Biomedical Sciences, University of Padua, 35122, Padua, Italy.; 7 Maria Cecilia Hospital, GVM Care & Research, 48033, Cotignola, Italy.

**Keywords:** extracellular nanovesicles, exosomes, miRNA, liver diseases, regenerative medicine.

## Abstract

Exosomes are nanosized extracellular vesicles secreted by all cell types, including canine adipose-derived stem cells (cADSCs). By mediating intercellular communication, exosomes modulate the biology of adjacent and distant cells by transferring their cargo. In the present work after isolation and characterization of exosomes derived from canine adipose tissue, we treated the same canine donors affected by hepatopathies with the previously isolated exosomes. We hypothesize that cADSC-sourced miRNAs are among the factors responsible for a regenerative and anti-inflammatory effect in the treatment of hepatopathies in dogs, providing the clinical veterinary field with an effective and innovative cell-free therapy. Exosomes were isolated and characterized for size, distribution, surface markers, and for their miRNomic cargo by microRNA sequencing. 295 dogs affected with hepatopathies were treated and followed up for 6 months to keep track of their biochemical marker levels. Results confirmed that exosomes derived from cADSCs exhibited an average diameter of 91 nm, and positivity to 8 known exosome markers. The administration of exosomes to dogs affected by liver-associated inflammatory pathologies resulted in the recovery of the animal alongside the normalization of biochemical parameters of kidney function. In conclusion, cADSCs-derived exosomes are a promising therapeutic tool for treating inflammatory disorders in animal companions.

## Introduction

Exosomes are extracellular vesicles that range in size from 30 nm up to 150 nm in diameter, comprised in a lipid bilayer and secreted by multiple cell lines. They can be found in a variety of biological fluids (including urine, plasma, and colostrum), and can also be isolated from the conditioned media of cells cultured *in vitro* 1-3. Exosomes play a major role in cell-cell communication both at a paracrine and systemic level. They are involved in either physiological processes (i.e., immune response, cell proliferation, and differentiation) or pathological ones (i.e., cancer progression, cardiovascular diseases) 1,4,5. Exosomes contain nucleic acids, proteins, lipids, and other metabolites that are secreted by donor cells and taken up by recipient cells.

In particular, exosomal microRNAs (miRNAs) have recently gained attention because of their ability to regulate gene expression 6-8. miRNAs are a class of single-stranded, non-coding RNA of approximately 22 nucleotides 6,9. miRNAs are mostly transcribed from DNA, processed into precursor miRNAs, and finally into mature miRNAs 6, 10. They are responsible for the alteration and degradation of a target mRNA transcript via binding with its 3' UTR, leading to the suppression of biological functions 11, 12. miRNAs expression can be correlated to several physio-pathological conditions 13,14.

In human medicine, great importance is currently given to the miRNA cargo of exosomes employed as diagnostic and prognostic biomarkers to diagnose a condition or to monitor its development before and after treatment - mainly concerning neoplastic malignancies 15. By contrast, veterinary medicine still requires data supporting the biomedical application of exosomes 16. Recent studies have shown how exosomes can be adopted in the framework of oncology and orthopedic diseases with positive results in animals carrying liver diseases 17,18, however, to our knowledge, literature is still lacking exosome application as a treatment for inflammatory diseases such as liver diseases in companion animals.

In the present study, liver hepatopathies were selected because the liver represents the organ that mostly shows great perspectives for the use of exosomes as regenerative strategy 19. As it is well known indeed, in healthy conditions liver cell turnover is very slow, but at the same time, after 70% partial hepatectomy the liver is the only organ whose size returns to the original extent in about two weeks 20. Despite this, in chronic and severe acute liver disease, this regenerative and replicative ability of the hepatic stem cells fails to regenerate the liver, as diffuse inflammation is present inducing increased production of matrix proteins, decreased matrix remodeling, progression to fibrosis, increase in the proportion of senescent hepatocytes (i.e., hepatocytes arrested at G1/S transition of the cell cycle) and hepatocyte death 21. In this context, dogs primarily present with parenchymal pathologies such as hepatitis with an estimated frequency of up to 12% in the general canine population 21-23. Considering this, in the present work we focused our attention on liver-inflamed diseases, to shift our previous study, on which we demonstrated that the use of mesenchymal stem cells in such pathology is a winning strategy 24, from a cell-based therapy to a cell-free therapy. To reach this aim we focused our research on the characterization of canine exosomes from adipose-derived mesenchymal stem cells (cADSC-exos) and the efficacy of their clinical application on dogs suffering from inflammatory conditions in the liver.

## Material and methods

### Ethics statement

The present study follows the guideline published in the Official Gazette the October 17, 2013 agreement “Guidelines concerning the minimum health requirements for the use of stem cells in veterinary medicine” (OJ General Series No. 277, 11-26-2013). The guideline applies to MSC Multipotent Stromal Cells that have not undergone relevant manipulation, prepared on a non-repetitive basis, for a product intended exclusively for autologous use, in a specific animal owned by legal and/or natural persons, subjected to their informed consent. To this view, only the informed consent was needed.

### Isolation and culture of canine ADSCs

Cells were isolated from the fat tissue of canine specimens. Briefly, biopsies were washed with PBS (Phosphate buffered saline, Aurogene, Rome, Italy), dissected, and digested with collagenase type II (Sigma-Aldrich, St. Louis, Missouri, USA) in Hanks' balanced salts solution (HBSS) with calcium and magnesium (EuroClone, Pero, Milan, Italy) for 3 hours at room temperature with intense shaking. The cellular fraction was pelleted and rinsed with PBS.

Cells were cultured in DMEM Dulbecco′s Modified Eagle′s Medium high glucose (Sigma-Aldrich, St. Louis, Missouri, USA) supplemented with 2% Antibiotic-Antimycotic Solution 100× (Sigma-Aldrich, St. Louis, Missouri, USA) and 10% Fetal Bovine Serum (FBS) (Thermo Fisher Scientific, Waltham, Massachusetts, USA) at 37 °C and 5% CO2. Cellular growth was observed via the Evos XL Core microscope (Invitrogen, Waltham, Massachusetts, USA).

### Flow cytometry of cADSCs

Cells growing in adherence were detached and resuspended in flow cytometry staining buffer (R&D Systems, Minneapolis, Minnesota, USA) to a final concentration of 1x10^6^ cells/mL, as in our previous work.^22^ cADSCs were incubated with the fluorescent antibodies CD90-APC, CD44-FITC, CD14-PE, CD45-FITC (Thermo Fisher Scientific, Waltham, Massachusetts, USA). Flow cytometry was carried out with Attune™ NxT Acoustic Focusing Cytometer (Life Technologies, Carlsbad, California, USA), and data were analyzed using the Attune NxT Soft-ware version 2.5.

### Exosome isolation

Cells were cultured in a suitable medium consisting of DMEM Dulbecco′s Modified Eagle′s Medium high glucose (Sigma-Aldrich, St. Louis, Missouri, USA) supplemented with 2% Antibiotic-Antimycotic Solution 100× (Sigma-Aldrich, St. Louis, Missouri, USA) and 10% exosome-depleted Fetal Bovine Serum (Thermo Fisher Scientific, Waltham, Massachusetts, USA) to avoid serum lipoprotein contamination. After 72 hours, exosomes were isolated using the Amicon® Ultra-15 Centrifugal Filter Units with Ultracel-100 regenerated cellulose membrane (cat. no. UFC910024, Millipore, Burlington, Massachusetts, USA); conditioned medium was centrifuged at 2000 rcf for 30 minutes at +4 °C, followed by washing with PBS at 2000 rcf for 30 minutes at +4 °C; eventually, exosomes retained by the filter were collected and stored at -20 °C.

### Tunable Resistive Pulse Sensing (TRPS) measurements

Exosomes were characterized in size and concentration via the qNano Gold (IZON Science LTD, Christchurch, New Zealand). Measurements were performed at a pressure of 20 mbar and normalized using 100 nm calibration particles. Data were analyzed and plotted with the qNano Control Suite Software.

### Scanning Electron Microscopy (SEM)

Samples were fixed with 2% glutaraldehyde in phosphate buffer at +4 °C, dehydrated with ethanol, and eventually sputter-coated with gold/palladium. Imaging was performed with the SEM Zeiss EVO 40 microscope (Zeiss, Oberkochen, Germany) at the Centro di Microscopia Elettronica (University of Ferrara, Ferrara, Italy).

### Protein quantification

Exosome protein quantification was performed using the Pierce™ BCA Protein Assay Kit (Thermo Fisher Scientific, Waltham, Massachusetts, USA), as per instructions of the manufacturer. Briefly, bovine serum albumin (BSA) was used to obtain a 9-points standard curve. 10 μL of each sample were pipetted, in duplicate, into a 96-well Falcon® culture microplate (Corning Inc., New York, USA). 200 µL of working reagent solution provided in the kit were added and incubated for 30 minutes at 37 °C. Finally, a multilabel plate reader (Victor 3, Perkin Elmer, Milan, Italy) was used to read the absorbance. Data are expressed as means ± SEM.

### Exosomes antibody array

The ELISA test of exosome-specific markers was performed with the Exo-Check™ Exosome Antibody Array (Systems Biosciences, Palo Alto, USA) according to the manufacturer's instructions. 8 known exosome markers were included: CD81, CD63, FLOT1, ALIX, ANXA5, EpCAM, TSG101 and ICAM1.

### Fluorescent Labeling and image acquisition

cADSC-exos were stained with the PKH26 Red Fluorescent Cell Linked Midi Kit for General Cell Membrane Labeling (Sigma-Aldrich, Saint Louis, USA). Exosomes were incubated with a staining solution of diluent C and PKH26 dye, followed by blocking with an equal volume of exosome-depleted FBS; to remove the excess staining, 10 mL of PBS were added and the solution was centrifuged at 37400 rcf for 30 minutes with an Ultracentrifuge Optima L-70 (Beckman Coulter Inc., Brea, USA), type 70 Ti rotor. The supernatant was removed, and the pellet was resuspended in 200 µL PBS. 20000 cADSCs were seeded on 13 mm glass slides and treated for 24 hours with the PKH26-stained cADSC-exos. Specimens were then fixed with PFA 4%, blocked with BSA 1%, and incubated with Hoechst fluorescent dye (Sigma-Aldrich, St. Louis, USA) to stain cell nuclei and Alexa Fluor™ 488 Phalloidin (Sigma-Aldrich, St. Louis, USA) to stain actin filaments. Images were acquired with the Zeiss Axiovert 200M Fluorescence Microscope (Zeiss, Oberkochen, Germany) equipped with a 63x oil objective.

### Exosomal RNA extraction

Exosomal RNA was extracted using the Cell Culture Media Exosome Purification and RNA Isolation Mini Kit (cat. no. 60700, Norgen Biotek Corp., Thorold, Canada). RNA samples were stored at -80°C.

### microRNA (miRNA) sequencing

miRNA-seq libraries were generated using the QIAseq miRNA Library Kit (Qiagen; Hilden, Germany) and sequenced using NovaSeq 6000 (Illumina, San Diego, USA) in 2x150 paired-end mode. miRNAs found in the specimens were identified using the QIAseq miRNA-NGS data analysis software with single-end read as read type and 75 cycles in Read 1. Datasets from miRNA sequencing were analyzed with the Qiagen Ingenuity Pathway Analysis (IPA) software.

### Statistical Analyses

All experiments were performed in triplicate, and each experiment was performed independently three times. *In vitro* results were expressed as mean ± standard deviation (SD). *In vivo* results were expressed as mean ± SEM.

### Blood sample collection and detection of blood biochemical markers

Blood samples were obtained from the jugular vein of dogs of the Maltese, Akita, Basset Hound, Beagle, Bernese Mountain Dogs, Cavalier King Charles Spaniel, Chihuahua, Shih tzu, Siberian Husky, Mastiff, Cane Corso, and Miniature Schnauzer dog breeds. The blood of canine donors was collected before the injection and after 30 and 180 days, then stored at -20°C for analysis using commercial sandwich ELISA kits (Thermo Fisher Scientific, Waltham, Massachusetts, USA). Alanine Transaminase (ALT), Alkaline Phosphatase (ALP), Alanine Aminotransferase (AST), Gamma-Glutamyl Transferase (GGT), Total Serum Bile Acid (TSBA), Blood Urea Nitrogen (BUN). Optical density values at 405 nm were measured using a multilabel plate reader.

### Injection of autologous cADSCs-derived exosomes

295 dogs with severe degenerative hepatopathy were included in this study. Dogs with cancer in the liver or other organs were excluded. Thrombosis of the hepatic vein was another criterion of exclusion. Dogs were selected following the American College of Veterinary Internal Medicine (ACVIM) consensus statement on the diagnosis and treatment of chronic hepatitis 25. For the injection, exosomes (0.2 mg of protein/mL per kg) suspended in 2 mL PBS were transfused through the peripheral vein. Dogs underwent two injections of autologous exosomes at 30 and 180 days.

### Specimen collection and cytopathological examinations

Hepatic cytology specimens were obtained by ultrasound-guided fine-needle capillary sampling without aspiration before the first exosome injections and 30 days after the second treatment. Smears were air-dried, stained using the Diff-quick stain (Merck, Darmstadt, Germany), and then imaged under a light microscope.

## Results

### Characterization of canine ADSCs

**Figure 1:**
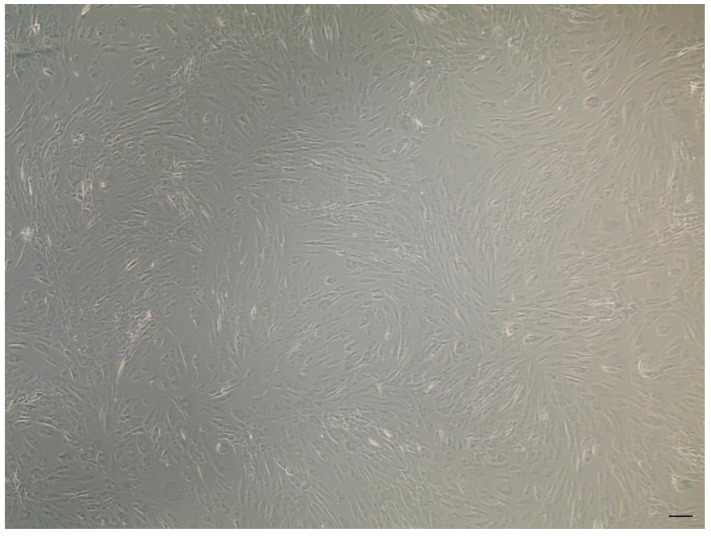
Canine ADSC characterization. cADSCs showed their typical elongated morphology. Scale bar is 20 µm. Images were taken with the Evos XL Core microscope (Invitrogen, Waltham, Massachusetts, USA) equipped with a 4x objective.

**Figure 2:**
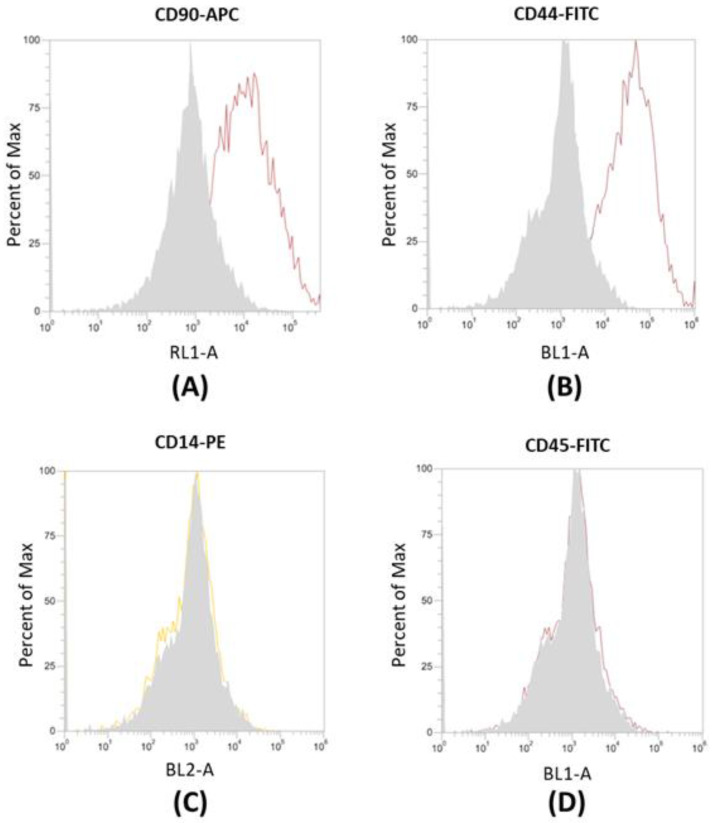
Flow cytometry analysis of surface markers in cADSCs. (A-B) cADSCs are positive for CD90-APC and CD44-FITC. (C-D) cADSCs are negative for CD14-PE and CD45-FITC. Measurements were taken with the Attune™ NxT Acoustic Focusing Cytometer (Life Technologies, Carlsbad, California, USA).

Canine ADSCs (cADSCs) were isolated and maintained in culture in tissue culture flasks, exhibiting their characteristic fusiform, fibroblastic-like morphology (Figure 1). cADSCs were positive for MSC-selective surface markers CD90-APC and CD44-FITC (Figure 2A and Figure 2B), and negative for CD14-PE and CD45-FITC (Figure 2C and Figure 2D).

### Characterization of canine ADSC-derived exosomes (cADSC-exos)

**Figure 3:**
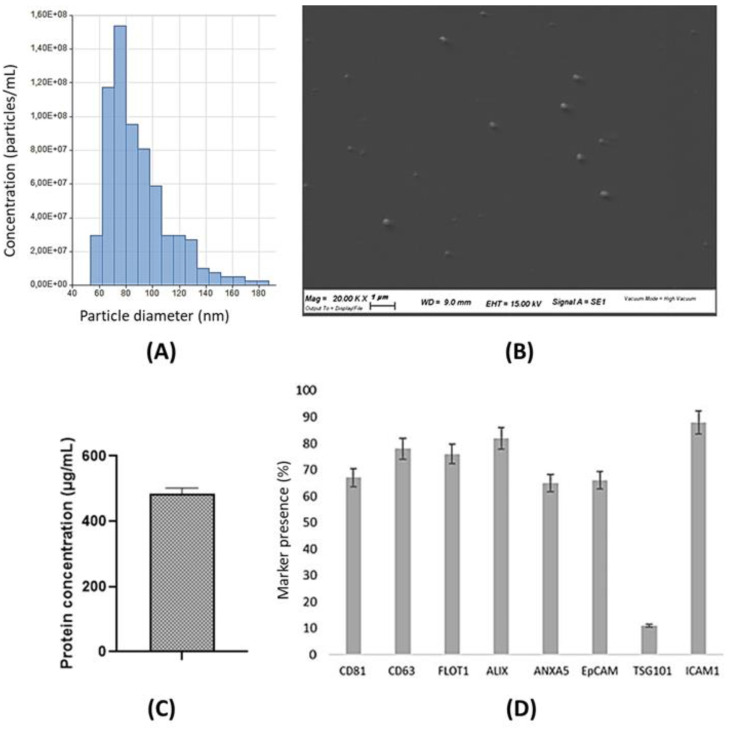
cADSC-exo characterization. (A) The histogram shows the size distribution of cADSC-exos evaluated by Tunable resistive pulse sensing. The mean diameter is 91 nm (St. Dev ± 28.7), while the average concentration is 6.62x10^8^ particles/mL. (B) SEM images confirm the exosome diameter. Images were taken with the SEM Zeiss EVO 40 microscope (Zeiss, Oberkochen, Germany) at a 20.000x magnification. (C) cADSC-exos show a protein concentration of 483.9µg/mL (St. Dev ± 18.3). Results are the mean of 3 replicates. (D) cADSC-exos are positive for the specific markers MSC-derived exosomes CD81 and CD63. Results are expressed as percentages compared to positive control. Analyses were performed in triplicate.

The supernatant was collected from canine adipose-derived mesenchymal stem cells (cADSCs), and exosomes were isolated with a commercial kit as described in the Material and methods section. Exosomes were characterized using the Tunable Resistive Pulse Sensing (TRPS) technique, a single-particle technology that allows a precise and accurate measurement of exosome diameter. Exosome concentration was 6.62x10^8^ particles/mL, with an average diameter of 91 nm (Figure 3A). Exosome size was further confirmed by scanning electron microscopy (SEM) (Figure 3B). According to the BCA assay, the mean protein concentration of cADSC-exos is estimated at 483.9 µg/mL (Figure 3C). Protein content evaluation confirmed cADSC-exos positivity to CD81 and CD63, which are surface markers characteristic of MSC-derived exosomes (Figure 3D). Taken together, these data are in line with the Minimal information for studies of extracellular vesicles (MISEV) 26 and demonstrate that the conditioned medium of cADSCs is a source of exosomes.

### cADSC-exos interact with and are internalized by cADSCs

**Figure 4:**
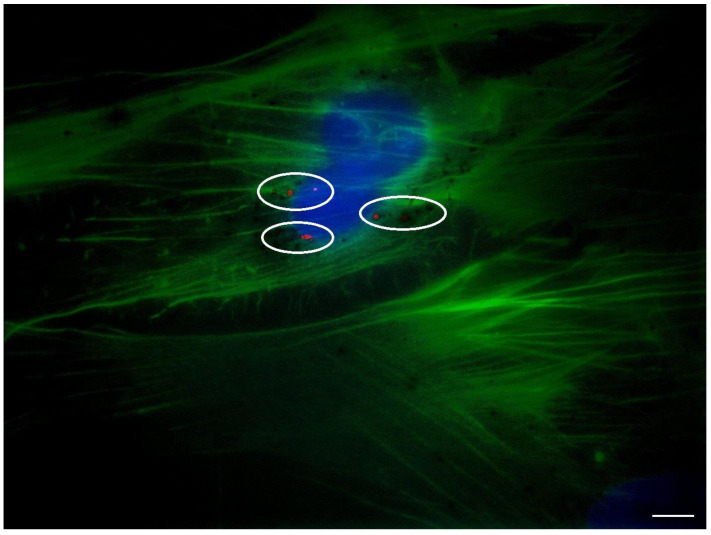
Internalization of cADSC-exos by cADSCs. PKH26-stained exosomes have been taken up by cADSCs and appeared in the cytoplasmic region of cells as red spots. Images were acquired with the Zeiss Axiovert 200M Fluorescence Microscope (Zeiss, Oberkochen, Germany) equipped with a 63x oil objective. Cell nuclei are stained in blue; actin filaments are stained in green; exosomes are stained in red. Scale bar is 20 µm.

cADSC-exos were stained with the PKH26 membrane dye, and after a 24-hour treatment, their interaction with cADSCs was evaluated via fluorescent microscopy. As shown in Figure 4, exosomes (highlighted in red and inside the circles) were taken up by cADSCs (whose nuclei are in blue and actin filaments in green) and detected in the cytoplasm.

### miRNA profile of cADSC-sourced exosomes and most abundant miRNAs

**Figure 5:**
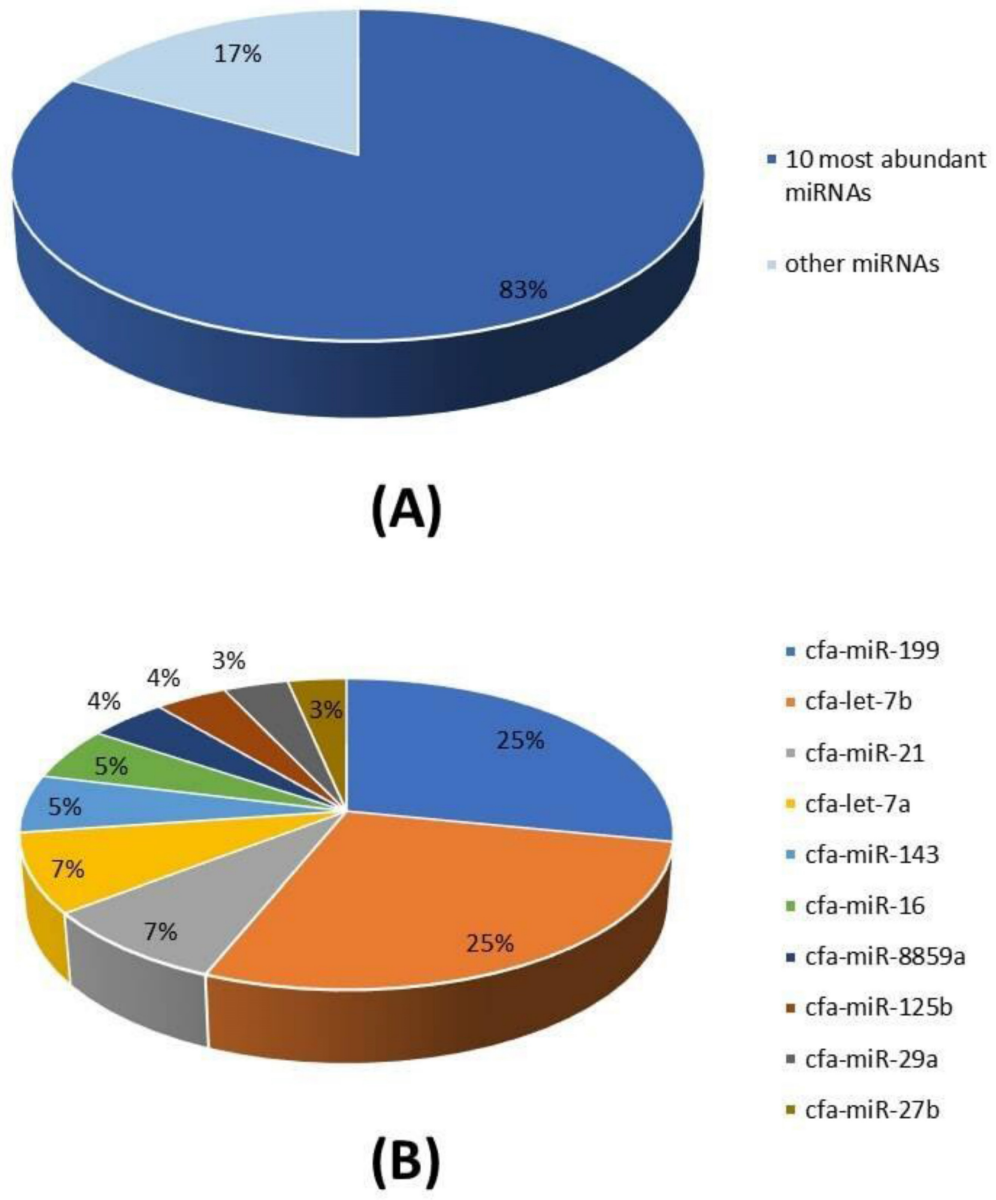
miRNA cargo of cADSC-exos. (A) The 10 most abundant miRNAs represent the 83% of the total miRNA cargo in cADSC-exos. (B) Distribution of the 10 top-ranked miRNAs. Analysis was performed in triplicate.

A total of 26 miRNAs was identified after microRNA sequencing (miRNA-seq) and the alignment of the individual transcripts to the *Canis lupus familiaris* genome (Table 1). The 10 most abundant miRNAs represent more than 80% of all detected miRNAs in cADSC-exos (Figure 5A) and are respectively: cfa-miR-199, cfa-let-7b, cfa-miR-21, cfa-let-7a, cfa-miR-143, cfa-miR-16, cfa-miR-8859a, cfa-miR-125b, cfa-miR-29a, cfa-miR-27b (Figure 5B).

### miRNAs expressed in mesenchymal stem cell-derived exosomes hold regenerative properties

Literature review revealed that human MSC-sourced miR-199, let-7b, miR-21, miR-125b, and mir-29a are responsible for regenerative effects by positively modulating angiogenesis, cell, proliferation, and tissue remodeling in several inflammatory conditions (Table 2).

### miRNA transcripts with regenerative properties are conserved between human and canine genome

The “microRNAviewer” web server was employed to investigate the homology of miRNA families among species.^35^ This tool showed that the miRNA transcripts of miR-199, let-7b, miR-21, miR-125b, and mir-29a are conserved between the human and canine (“cfa”) genomes, with a degree of conservation close or equal to 1 (Table 3).

### *In vivo* injection of autologous exosomes in dogs ameliorates clinical signs of liver disease

The selection of the dogs and the diagnostic tests, the treatment procedure, and the conduction of clinical research in hepatology followed the consensus statement on chronic hepatitis (CH) in dogs were based on the expert opinion of 7 specialists with extensive experience in CH 25. This is because the panel that identifies the diagnosis and treatment of CH in dogs is a complex process that requires the integration of clinical signs with clinical pathology, diagnostic imaging, and hepatic biopsy (Figure S1).

Clinical signs reported for dogs with CH are summarized in Table 4. Clinical signs typically are not specific - such as loss of appetite and lethargy. As reported in Table 4, the first injection of exosomes induces the recovery of two important clinical signs - decreased appetite and depression.

**Figure 6:**
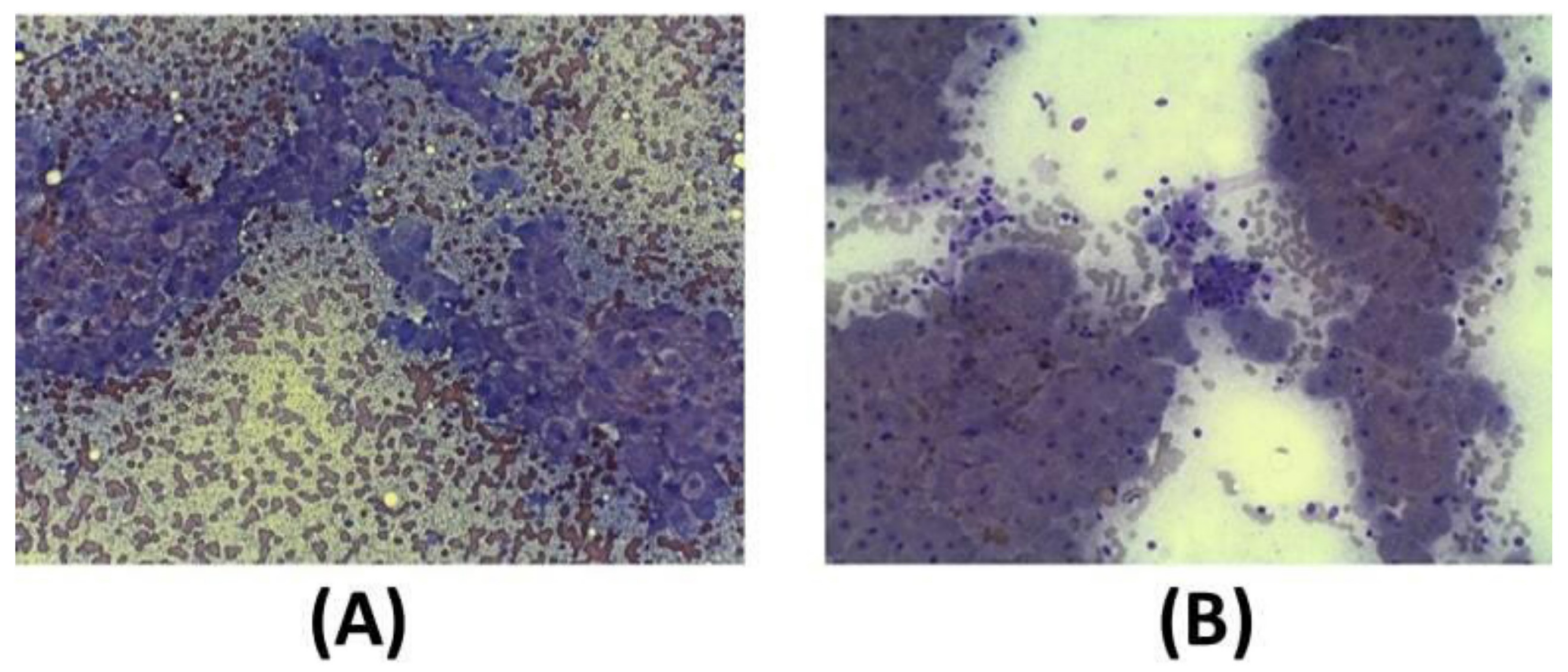
Diff-quick stain of cytological samples before and 30 days after the second exosome injection after 30 days. (A) Before exosome treatment, inflammatory processes are occurring with foamy macrophages and hepatic debris; red cells are also visible. (B) After exosome treatment, fibrosis and inflammation are strongly reduced.

In addition to clinical sign, cytological analysis in a small (12) group of dogs before the first exosome injections and 30 days after the second injection was performed. As reported in the guidelines, the key features of liver biopsy specimen interpretation include evaluation of inflammation (granulomatous, neutrophilic, lymphocytic, suppurative inflammation). Moreover, it is mandatory to analyze the extent and distribution of cell death (i.e., individual, massive, multifocal, centrilobular, and periportal cells), characterized by vacuolar shift. Often, fibrosis of the connective tissue is also present as septa formation and nodular regeneration of hepatocytes. Before exosome injections, hepatocytes show a round central nucleus, and the presence of inflammation process is demonstrated by the presence of neutrophils, small and medium activated lymphocytes, and foamy macrophages **(Figure 6A)**. After the second injection of exosomes (after 30 days), cytological images show that liver inflammation is declined, as well as the presence of spindle cells; furthermore, no sign of fibrosis is evident **(Figure 6B)**.

We were cautious since the primary concern for any hepatic sampling technique is post-procedural haemorrhage due to coagulation abnormalities related to the alteration of normal liver function. Risks of hepatic biopsy other than haemorrhage include anaesthetic complications, air embolism and pneumothorax (with laparoscopy), and infection. Hence, we used ultrasound to define the shift of portal hypertension. As reported in Table 5, portal hypertension strongly improves after exosome injections.

**Figure 7:**
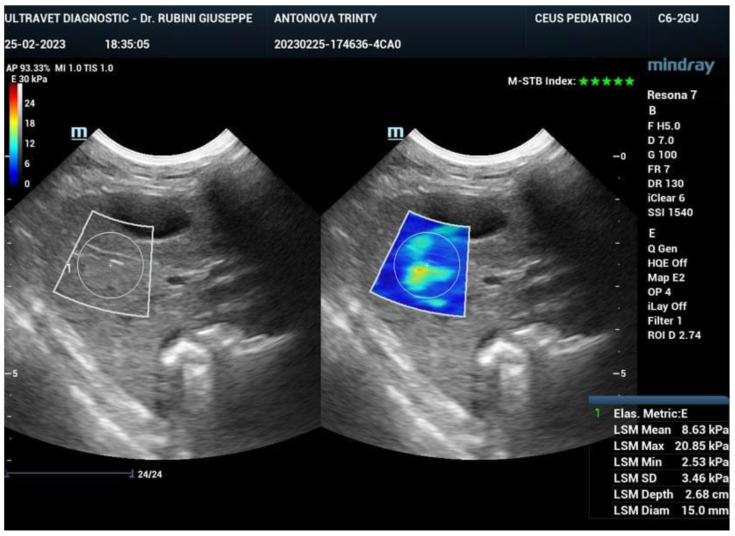
Sonoelastography of the canine liver before injection of autologous exosomes. Fibrotic tissue and altered portal vein blood flow are shown.

**Figure 8:**
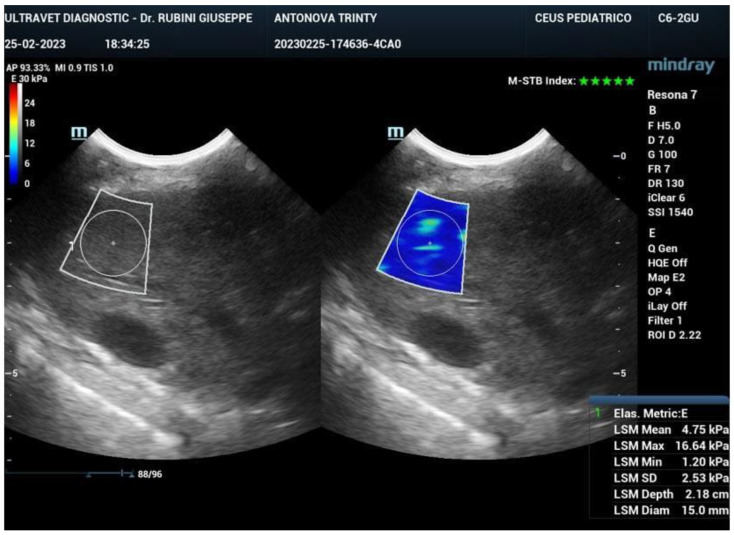
Sonoelastography of the canine liver 180 days after injection of autologous exosomes. No fibrotic tissue is visible, and the portal vein blood flow is restored.

Ultrasound elastography (also known as sonoelastography) was used to support the diagnosis and management of diffuse liver disease. Elastography can identify early-stage and advanced liver fibrosis and thus is a major application in clinical liver care. In addition, elastographic characterization of focal liver lesions and evaluation of clinically significant portal hypertension can potentially be clinically useful and are areas of active clinical research. Currently, the accepted liver fibrosis staging reference standard is the histopathologic evaluation of non-focal liver biopsy specimens. However, liver biopsy has some limitations: 1) liver biopsy is invasive, 2) liver biopsy is costly, and 3) inter-observer variability limits the clinical interpretability of liver biopsy samples. To this end, 34 dogs treated with exosomes were followed also by sonoelastography to evaluate the presence and reduction of fibrosis after 180 days from the first injection of exosomes. As reported in Figure 7, fibrotic tissue with alteration of blood flow (highlighted in the circle) is visible before the treatment. Figure 8 shows that the fibrotic event is no more present and the portal vein blood flow has recovered after 180 days from the first injection of exosomes.

Serum enzymology was performed to detect serum liver enzyme activities focalizing our attention on alanine transaminase (ALT) activity - which is the earliest indicator of CH, and alkaline phosphatase (ALP) activity - that occurs later in CH. Since the liver is the largest synthetic reserve for albumin synthesis, hypoalbuminemia has been detected. Together with these markers, gamma-glutamyl transferase (GGT), total serum bile acid (TSBA), blood urea nitrogen (BUN), and cholesterol levels were assessed. As reported in Table 6, all biochemical blood markers recovered 180 days after exosome treatment.

## Discussion

Mesenchymal stem cells (MSCs) are multipotent stem cells crucial for tissue regeneration and repair that can be isolated from different sources, including adipose tissue 36. In the last decade, MSCs have drawn attention because they possess some peculiar properties: 1) self-renewal, 2) multilineage differentiation, 3) secretion of trophic and growth factors, 4) immunomodulation, and 5) ease of amplification *in vitro* 36,37. Concerning dogs carrying liver diseases, adipose tissue can be easily collected during well-controlled routine procedures such as sterilization, without exposing the animal to additional surgeries. We already demonstrated that autologous adipose-derived mesenchymal stem cells (ADSCs) can be successfully employed as a treatment for hepatic disorders in the veterinary field 24, however, exosomes could represent an alternative cell-free therapy as they retain the same features of the cells they originate from. Assessing the proper isolation of cADSCs was a fundamental first step in our research, and we also confirmed the results obtained from our previous study 24. Indeed, flow cytometry confirmed the positive expression of CD90-APC and CD44-FITC surface markers and negativity to CD14-PE and CD45-FITC in the isolated cADSCs (Figure 2).

Adipose-derived mesenchymal stem cells (ADSCs) are not only easy to expand *in vitro*, but they also secrete a considerable amount of exosomes 15,37. Furthermore, the immunomodulatory and regenerative properties of MSCs are preserved in MSC-derived exosomes 37, making these cells an ideal source to obtain exosomes. Vesicle characterization is essential to univocally identify exosomes. In our work, cADSC-exos were analyzed through an ELISA kit (Figure 3). Additionally, exosome size distribution was measured via the TRPS approach and SEM imaging (Figure 3).

Exosomes act as messengers in the process of intercellular communication at a paracrine and endocrine level 5, interacting with cells (as established in Figure 4) and transferring their cargo to recipient cells - thus changing their phenotype or regulating gene expression 14. The exosome loading is sorted by ESCRT-dependent and independent mechanisms, and it is influenced by the parental cell and environmental characteristics 14,38,39. Nevertheless, the final content of exosomes includes proteins, lipids, nucleic acids, and other metabolites 2.

Concerning nucleic acids, we focused on the miRNome of exosomes, as this family of molecules plays a role in pathophysiological conditions, targeting specific messenger RNAs (mRNAs) and determining their degradation or translation repression. miRNA-seq from cADSC-derived exosomes determined that 26 miRNAs were associated with the *Canis lupus familiaris* genome, as identified by the prefix “cfa” (Table 1). As reported in the “Results” section, the 10 top-ranked miRNAs accounted for 83% of all the miRNAs detected (Figure 5).

To put our findings into context, literature research was conducted (Table 2). It is acknowledged that exosomes derived from mesenchymal stem cells reduce inflammatory processes while providing a positive impact on collagen deposition, angiogenesis, and cell proliferation in a plethora of inflammatory conditions 40-42. In particular, miR-199 promotes liver regeneration by enhancing hepatocyte proliferation while decreasing their apoptosis 27. MSC-sourced let-7b decreased renal inflammation through the polarization of renal macrophages to the M2 phenotype while reducing inflammatory cytokine production 28. miR-21 plays a role in controlling the stem cell niche at the site of injury, promoting cell proliferation and angiogenesis through the activation of protein kinase B and extracellular signal-regulated kinase 29,30. Moreover, miR-21 mediates the maturation and migration of infiltrating dendritic cells and decreases inflammation through the NF-kB pathway in kidney inflammation 31,32. miR-125b plays a role in renal angiogenesis by partially inhibiting p53 signaling, improving tubular epithelial cell proliferation, and limiting their apoptosis 33. Wang and colleagues demonstrated that exosomal miR-29a limits renal fibrosis in a unilateral ureteral obstruction mice model via the downregulation of the fibrotic-related proteins YY1, TGF-β1, and TGF-β3 34. Overall, MSC-derived exosome miRNome shows immunosuppressive and protective features that relieve inflammatory conditions (Table 2) 40.

Interestingly, miR-199, let-7b, miR-21, miR-125b, and miR-29a fall under the category of mitomiRs, which are miRNA transcripts specifically associated with mitochondrial metabolism or enriched in this distinct organelle. Zheng and colleagues analyzed the expression of mitomiRs in purified mitochondrial fractions of MSC and from whole extracts of MSC, identifying several mitomiRs which regulate several mitochondrial functions 43.

Surprisingly, many software and online tools used for functional analysis of OMICs data or literature research are not equipped with databases other than human. To overcome this issue, we investigated the homology between the 10 most abundant miRNAs in the dog (“cfa”, *Canis lupus familiaris*) with the corresponding miRNA of the human species (“hsa”, *Homo sapiens*), sure in the knowledge that many miRNA families, as well as their targets, are conserved among different animal species 44-46. The miRviewer software confirmed the conservation between dog and human of cfa-miR-199, cfa-let-7b, cfa-miR-21, cfa-miR-143, cfa-miR-125b, cfa-miR-29a, and cfa-miR-27b, with a degree of conservation close or equal to 1 (Table 3).

Taken together, data from the current research suggested that cADSC-exos could be used as a cell-free therapy for the treatment of CH in dogs. Such disease is well characterized in human medicine from a pathological and clinical perspective 24,45,47. Noteworthy, Volk and colleagues emphasized that humans share similarities with dogs more than other animal species in terms of anatomy, physiology, and similar disease onset 24,48. Consequently, human liver diseases resemble the ones occurring in animal species and thus results from human research may be translated to the veterinary field 24,48,49.

In light of these considerations, 295 dogs affected with hepatopathies were treated with autologous exosomes following the recommendation of the ACVIM consensus guideline 25. Fibrosis, inflammation, and liver function activity levels were first defined through histology, imaging technology, and biochemical analyses; then, the progress of clinical signs and biochemical markers was assessed 30 and 180 days after the first injection of exosomes. As reported, the first positive clinical evidence is represented by the recovery of appetite and depression state of dogs (Table 4), followed by the recovery of fibrosis, inflammation, and liver parameters such as ALT, AST, ALP, GGT, and TSBA (Table 6).

## Conclusion

Treatment of hepatopathies and CH in dogs should target the causative agent, unfortunately, this liver disease is often idiopathic. Concerning autologous exosome treatment, it can be indicated with non-specific hepatoprotective agents with or without a trial of immunosuppressive treatment as also suggested by ACVIM consensus. In this view, exosomes derived from canine adipose-derived stem cells have proven to be a valid therapeutic tool for the treatment of inflammatory liver disease in dogs, and possibly in the veterinary field in general.

## Figures and Tables

**Table 1:** Summary of miRNAs detected in exosomes derived from cADSCs. The reads are the average of three replicates. “cfa”: *Canis lupus familiaris*.

miRNA	Mean reads		miRNA	Mean reads
cfa-miR-199	320		cfa-miR-221	22
cfa-let-7b	319		cfa-miR-378	21
cfa-miR-21	96		cfa-miR-148a	18
cfa-let-7a	94		cfa-let-7c	16
cfa-miR-143	69		cfa-miR-122	16
cfa-miR-16	63		cfa-miR-193a	16
cfa-miR-8859a	51		cfa-miR-25	14
cfa-miR-125b	47		cfa-miR-320	13
cfa-miR-29a	42		cfa-miR-145	12
cfa-miR-27b	38		cfa-miR-28	12
cfa-miR-423a	28		cfa-miR-26b	11
cfa-let-7f	25		cfa-miR-203	9
cfa-let-7e	24		cfa-miR-196a	7

**Table 2:** Studies demonstrating the positive outcome of exosomal miRNA transfer in inflammatory conditions. The table summarizes the major findings on miR-199, let-7b, miR-21, miR-125b, and mir-29a in the treatment of inflammatory conditions.

Intervention field	Outcome	Major findings	miRNA transferred	References
Liver regeneration	Liver regeneration and hepatocyte proliferation	↑ hepatocyte proliferation ↓ hepatocyte apoptosis	miR-199	27
Renal inflammation	Renal protection	↑ immunosuppressive M2 phenotype in renal macrophages ↓ production of inflammatory cytokines	let-7b	28
Acute inflammation at the site of injury	Stem cell niche maintenance	↑ multiple cellular proliferation ↑ blood vessel formation at the site of injury	miR-21	29,30
Renal inflammation	Renal protection	↓ NF-kB activity ↓ reduced maturation and function of infiltrating dendritic cells	miR-21	31,32
Ischemic acute kidney injury	Tubular epithelial cells maintenance	↓ tubular epithelial cells apoptosis by repressing p53 expression	miR-125b	33
Unilateral Ureteral Obstruction	Kidney damage attenuation	↓ renal fibrosis	miR-29a	34

**Table 3:** Conservation of miRNA transcripts between canine and the human genome. The conservation grade among canine (“cfa”, *Canis lupus familiaris*) and human (Homo sapiens) genomes for the miRNA of interest is between 0.98 and 1. The analysis was performed with the online tool microRNAviewer (miRviewer).

*Canis lupus familiaris* genome	Degree of conservation
cfa-miR-199	0.98
cfa-let-7b	0.98
cfa-miR-21	1
cfa-let-7a	0
cfa-miR-143	1
cfa-miR-16	0
cfa-miR-8859a	No degree
cfa-miR-125b	1
cfa-miR-29a	1
cfa-miR-27b	0.99

**Table 4:** Clinical signs of dogs suffering from CH before and 30 days after autologous exosome treatment. CH is mainly characterized by non-specific symptoms; autologous treatment injection resulted in the recovery of such symptoms, notably decreased appetite and lethargy/depression after 30 days.

Clinical signs before exosome treatment	Percentage (%) of dogs	Clinical signs 30 days after treatment	Percentage (%) of dogs
Decreased appetite	68	Recovery	96
Lethargy/Depression	63	Recovery	98
Icterus	37	Recovery	65
Ascites	35	Recovery	34
Vomiting	27	Recovery	73
Diarrhea	21	Recovery	12
Hepatic Encephalopathy	8	Recovery	81
Melena	5	Recovery	45
Abdominal Pain	2	Recovery	56

**Table 5:** Clinical signs related to portal hypertension. All the parameters are improved 30 and 180 days after treatment with autologous exosomes.

Clinical signs of portal hypertension	Percentage (%) of dogs	Improvement (%) 30 days after treatment	Improvement (%) 180 days after treatment
Decreased velocity of portal vein blood flow	83	25	42
Hepatofugal flow in the portal vein	76	21	65
Portal vein/aorta ratio ≤ 0.65 in the absence of a single congenital portosystemic shunt	81	27	64

**Table 6:** Biochemical blood markers panel of serum enzymes in liver disease. The variation of parameters before, and after exosome treatment (30 and 180 days) is reported in comparison to data from literature.

Parameter	Percent (%) change in literature	Variation before exosome treatment	Variation 30 days after treatment	Variation 180 days after treatment	Number of dogs
Increased ALT	85+/-16	81+/-15	74+/-12	44+/-15	63
Increased ALP	84+/-19	87+/-13	72+/-14	42+/-12	63
Increased AST	78+/-10	69+/-14	52+/-9	46+/-7	45
Increased GGT	61+/-12	66+/-15	56+/-18	32+/-11	45
Increased TSBA	75+/-14	72+/-11	62+/-13	54+/-13	63
Decreased BUN	40+/-29	36+/-12	31+/-10	21+/-14	23
Decreased Albumin	49+/-19	62+/-18	51+/-15	22+/-19	94
Decreased Cholesterol	40+/-12	36+/-23	32+/-13	18+/-11	23

Abbreviations: ALT, Alanine Transaminase; ALP, Alkaline Phosphatase; AST, Alanine Aminotransferase; GGT, Gamma-Glutamyl Transferase; TSBA, Total Serum Bile Acid; BUN, Blood Urea Nitrogen.

## Abbreviations

ACVIM: American College of Veterinary Internal Medicine
ALIX: ALG-2-Interacting protein X
ALP: Alkaline Phosphatase
ALT: Alanine Transaminase
ANOVA: Analysis Of Variance
ANXA5: Annexin A5
AST: Alanine Aminotransferase
BSA: Bovine Serum Albumin
BUN: Blood Urea Nitrogen
cADSCs: canine Adipose-Derived Stem Cells
cADSCs-exos: canine exosomes from Adipose-Derived mesenchymal Stem Cells
CH: Chronic Hepatitis
DMEM: Dulbecco′S Modified Eagle′S Medium
EpCAM: Epithelial Cell Adhesion Molecule
FBS: Fetal Bovine Serum
FLOT1: Flotillin 1
GGT: Gamma-Glutamyl Transferase
GM130: cis-Golgi Matrix protein 130kD
HBSS: Hanks' Balanced Salts Solution
ICAM: Intercellular Adhesion Molecule 1
IPA: Ingenuity Pathway Analysis
miRNA(s): microRNA(s)
miRNA-seq: microRNA sequencing
MISEV: Minimal Information for Studies of Extracellular Vesicles
PBS: Phosphate Buffered Saline
SEM: Scanning Electron Microscopy
TSBA: Total Serum Bile Acid
TSG101: Tumor Susceptibility Gene 101
TRPS: Tunable Resistive Pulse Sensing
