# Supplementary material for: Adipose-derived stem cell exosomes act as delivery vehicles of microRNAs in a dog model of chronic hepatitis
Source: Nanotheranostics. 2024 Mar 9;8(3):298–311. doi: 10.7150/ntno.93064 (PMC10988209; doi:10.7150/ntno.93064)
Supplement: Supplementary file 1 — Supplementary figures. [file ntnov08p0298s1.pdf]

Supplementary information

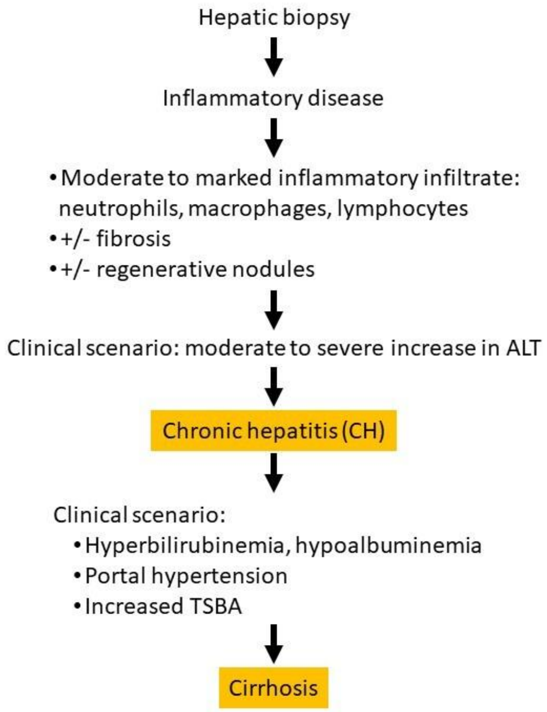

Figure S1. Panel for the diagnosis of liver diseases. Primary chronic inflammatory hepatopathies in dogs are usually accompanied by hepatocyte necrosis/apoptosis as well as different degrees of fibrosis. The integration of clinical signs and pathology, diagnostic imaging, and hepatic biopsy is required.
